# Regulatory mechanisms, prophylaxis and treatment of vascular leakage following severe trauma and shock

**DOI:** 10.1186/s40779-017-0117-6

**Published:** 2017-03-15

**Authors:** Chen-Yang Duan, Jie Zhang, Hui-Ling Wu, Tao Li, Liang-Ming Liu

**Affiliations:** State Key Laboratory of Trauma, Burns and Combined Injury, Second Department of Research Institute of Surgery, Daping Hospital, Third Military Medical University, Chongqing, 400042 China

**Keywords:** Clinical critical diseases, Vascular leakage, Vascular permeability, Shock, Sepsis

## Abstract

Vascular leakage, or increased vascular permeability, is a common but important pathological process for various critical diseases, including severe trauma, shock, sepsis, and multiple organ dysfunction syndrome (MODS), and has become one of the most important causes of death for intensive care units (ICU) patients. Currently, although there has been some progress in knowledge of the pathogenesis of these vascular disorders, the detailed mechanisms remain unclear, and effective prophylaxis and treatment are still lacking. In this study, we aimed to provide a review of the literature regarding the regulatory mechanisms and prophylaxis as well as the treatment of vascular leakage in critical diseases such as severe trauma and shock, which could be beneficial for the overall clinical treatment of vascular leakage disorders.

## Background

Capillary leak syndrome (CLS) refers to a series of syndromes with clinical manifestations of serious tissue edema, such as severe hypoproteinemia, hypovolemia and hypoperfusion, which is caused by the massive leakage of plasma proteins and intravascular fluid due to injuries to the vascular endothelium as well as increased vascular permeability [1]. The high mortality of CLS patients is believed to be associated with the non-specific clinical manifestations and the rapid progression of these disorders in the acute-onset phase [2]. Dhir et al. [3] found that the 5-year survival rate in CLS patients is approximately 70% and that 75% of the deaths occur in the acute-onset phase [4]. In addition, research has indicated that the 10-year mortality of CLS patients is approximately 34%, while deaths that occur in the intensive care units (ICU) are caused by both the acute onset and the complications of CLS, which account for 80% of the total mortality [5].

Under normal physiological conditions, water and electrolytes could pass through the capillary wall into the interstitial space instead of the plasma albumin, while proteins with molecular weight larger than 200 kD, or even 900 kD in some particularly severe circumstances, could also pass through the wall into the interstitial space. This physiological event occurs in cases of severe trauma, sepsis, cardiopulmonary bypass surgery (especially in infant cardiopulmonary bypass surgeries), reperfusion injury, venomous snake bites, acute lung injury, acute respiratory distress syndrome (ARDS), burns and drug toxicity (such as recombinant interleukin-2 and docetaxel). In certain drug toxicities, the mononuclear-phagocyte system, endothelial cells and neutrophils are excessively activated, resulting in the release of inflammatory cytokines and immune reactions, in which then produces injuries to the capillary endothelium, broken intercellular junctions and vascular leakage [6], which ultimately causes CLS. The major adverse effects of CLS include alveolar edema, restricted gas exchange and hypoxia, all of which aggravate injuries to the capillary endothelium cause edema in major organs such as the brain, the heart, the liver and the kidneys with the structures and functions that are damaged and that ultimately result in multiple organ dysfunction syndromes (MODS). Once that happens, the patient’s condition becomes more serious and includes higher risk for death [7]. Therefore, investigating the pathogenesis of vascular leakage or increased capillary permeability is of great significance for the prophylaxis and treatment of CLS. In this review, we will focus on newly identified regulatory mechanisms, and the prophylaxis and treatment of vascular leakage in critical clinical diseases such as shock.

## Pathways and regulatory mechanisms of vascular leakage

The vascular endothelium is composed of monolayer cells on the surface of vascular lumen and on the extracellular matrix in the deep layer. The vascular endothelium is a semi-permeable barrier between the vascular wall and the blood and controls the exchange of macromolecules and liquids between the interstitial fluid and blood. Generally, the occurrence of vascular leakage occurs through one of two pathways: the paracellular pathway, which refers to material that is diffused by passive transport through gap junction communication channels that are formed by intercellular junctions to the adjacent cells [8]; or the transcellular pathway, which refers to macromolecules that are transferred out of the vessel by endothelial cells instead of the intercellular junctions [9]. Although these two pathways can simultaneously exert synergistic or individual effects, previous quantitative analysis has confirmed that the paracellular pathway is the preferable pathway in cases of vascular leakage [10].

### Paracellular pathway

The paracellular pathway, also known as the interendothelial junction, refers to endothelial cells that are stimulated by an endogenous or an exogenous material. This stimulation of the paracellular pathway generates a series of variations in signal pathways to widen the intercellular gap by regulating the contraction of the endothelial cytoskeleton or by altering the intercellular junctions, which results in increased vascular permeability. There are three main types of endothelial cell junctions: tight junctions, adherens junctions between the cells, and adherens junctions between the endothelial cells and the basement membranes. Since the structure and protein composition of the tight junction and the adherens junction of endothelial cells were recently introduced in a summary report [11], we will mainly discuss the effects that these two kinds of junctions have in cases of vascular leakage.

#### The effect of the tight junction in vascular leakage

Tight junction proteins include the junction adhesion molecule (JAM), the occludin, the claudin, the zona occludens (ZO) and the cingulin. The JAM-1 can secure the stability of the tight junction and can regulate the permeability of the endothelial barrier by potentially mediating the construction of the reticular structure of the macromolecular complexes of the plasma membrane. Orlova et al. [12] found that the JAM plays an important role in regulating the functions of endothelial actin and myosin, (i.e., when the *JAM* gene is knocked off, the level of actin and the activity of myosin light chain phosphatase (MLCP) decreases, which leads to an increase in the level of phosphorylated myosin light chains and the formation of actin stress fibers. Occludin is not only a major composition of the tight junction but can also regulate the functions of the tight junction. Occludin can regulate the formation and decomposition of the tight junction and can maintain the integrity of the tight junction by adjusting the activity of the transmembrane proteins (actin and myosin) through the protein kinase C (PKC), the mitogen-activated protein kinase (MAPK) and the myosin light chain kinase (MLCK). Wong et al. [13] has shown that only phosphorylated occludin can combine with the tight junction. For occludin phosphorylation, serine/threonine are usually phosphorylated at the carboxyl terminal. Most of the phosphorylated occludin is distributed in the tight junctions, while the occludin and few phosphorylated residues is mainly distributed in the basement membrane of the cytoplasm [14]. Blasig et al. [15] found an increase in the transport of uncharged molecules by the tight junction barrier after excision of the carboxyl terminals on the *occludin* gene because of cell transfection. However, no variation has been found in trans-epithelial electrical resistance (TEER) with an increased permeability of the tight junction, which indicates that the increased permeability of the barrier is caused by an interruption of the mutual effect between tight junction proteins, instead of an increase in the TEER.

There are 24 molecules with highly homologous sequences in the claudin family that are named claudin −1 to −24 and that have molecular weight ranging from 20 to 27kD [16]. Binding sequences, located in the C-terminal of all claudins, can directly incorporate other tight junction proteins in the cytoplasm [17], such as ZO-1, ZO-2, ZO-3, which are proteins with PDZ domains as well as associated PALS-1 tight junction proteins. The mutual effect between ZO-1 and ZO-2 could lead to an indirect interaction between claudins and actin [18]. The dense band, composed of claudin polymers, could accelerate the formation of barriers. Van Itallie et al. [14] found that the phosphorylated S208 locus on the C-terminal of the cytoplasm of claudin-2 can influence the location of claudin-2 without changing the binding with ZO-1 or ZO-2. Recent research has confirmed that the extracellular domain of claudin-1 can not only affect the assembly of the tight junction but can also suppress its barrier functions [19]. Interestingly, this domain of claudin-1 is closely connected to occludin, which suggests that there might be a direct mutual effect between these two proteins, and this mutual effect is expected to be confirmed in future studies.

ZO-1 and ZO-2, which are two intermediate connectors, can closely bind the claudin, the occludin, and the JAM with actin and is also believed to be one of the key functions of the tight junction. Phosphorylation of the ZO-1 protein is intimately associated with the location of the intercellular tight junction and the permeability of cells, and excessive phosphorylation of the ZO-1 protein can relax the binding between occludin, leading to a decrease in function. In cells that lack the *ZO*-1/*ZO*-2 genes, the content of tight junction-related proteins (such as claudin) is relatively low [20], and the functions of the tight junction are also affected; however, the expression of tight junction-related proteins increases and the functions of the tight junction return to normal when the cells are transfected with *ZO*-1/*ZO*-2 genes. According to the latest research, Wang et al. [21] found that blood-brain barrier injuries caused by microwave irradiation were also associated with the decomposition of the tight junction and decreased function of the *ZO*-1 gene. Tyr-phosphorylation of occludin occurs because of microwave irradiation, which weakens the mutual effect between the occludin and the ZO-1 and results in a widened gap and a decomposition of the tight junction. Further studies have revealed that this process might produce these results by activating the vascular endothelial growth factor/fetal liver kinase 1 - extracellular signal-regulated kinase (VEGF/Flk-1-ERK) pathway.

#### The function of adherens junctions between cells in cases of vascular leakage

The adherens junction is one of the intercellular junctions results from the interaction between cadherin and the adjacent cellular membrane. Cadherin can bind with catenin, a kind of cytoplasmic protein, which is further connected to the cytoskeleton complexes (i.e., the actin filament or the microtubules) to stabilize the intercellular junctions [22]. In particular, vascular endothelial-cadherin (VE-cadherin) refers to the endothelial cadherin that is specifically expressed by vascular endothelial cells. As the most important adhesive composition of endothelial adherens junctions, VE-cadherin can exert its functions only by way of the VE-cadherin-catenin complex, which is composed of VE-cadherin, α-catenin, β-catenin and P120-catenin, etc. The VE-cadherin-catenin complex is also mediated by the targeted molecules of substances that can increase microvascular permeability [23, 24]. Multiple substances, such as VEGF, TNF-α, platelet activating factor, and thrombin or histamine, can induce the tyrosine phosphorylation of VE-cadherin, α-catenin and β-catenin to increase the vascular permeability [25, 26]. In EDTA-treated endothelial cells, a decrease in binding activity is seen between the VE-cadherin and the cytoskeleton [27, 28], which indicates that Ca^2+^ could protect the VE-cadherin from being hydrolyzed by proteolytic enzymes.

It should be noted that the functions of tight junctions also depend upon the integrity of intercellular adherens junctions. Caused by removal of extracellular Ca^2+^, loosened cables in adherens junctions will open the tight junctions [29]. However, Maiers et al. [30] indicated that by blocking the binding between ZO-1 and α-catenin, the stability of the adherens junctions was not affected, but damaged the endothelial barrier functions and destroyed the assembly with variations in ZO-1 motility and the structure of the actin cytoskeleton. Thus, the results suggest that binding between ZO-1 and α-catenin might be a new coupling mechanism for adherens junctions in the endothelial barrier.

### Transcellular pathway

Macromolecular substances are transferred out of the vessels through endothelial cells, instead of through the intercellular gap. Feng et al. [31] found that plasma proteins and other macromolecular substances can be diffused out of a morphologically intact capillary without any intercellular gaps. In addition, they also found alveoli with vesicles in the capillary endothelial membrane in ultrathin sections that were 14 nm in height, which could have been caused by pinocytosis. Macromolecular tracers can be rapidly diffused out of the vessel from the capillary vein through vesicular-vacuolar organelles (VVOs) [32, 33]. The protuberance, or small cavity formed by the cytoplasm of the capillary endothelial cells, is possibly associated with the regulation of focal blood velocity and flow and the exchange, synthesis, release, transformation of material and the inactivation of active substance. The angiotensin converting enzyme also exists in this small cavity. In addition, there are various proteins and enzymes distributed on the endothelial cells that express many receptors with diversified functions and structures. In addition to the vesicle transporter, aquaporin (AQP) on the endothelial membrane has been found to participate in the transcellular pathway.

#### Aquaporin

It had been long been believed that water could not pass through the membrane due to the hydrophobic property of the lipid bilayer in the membrane. However, it has been recently found that a 28 kD protein family on the membrane, namely, the AQP protein, that has a structure like other channel proteins, can adjust the transcellular permeability of water. The basic function of AQP is to mediate the transcellular transport of free water molecules. The major difference from other ion channels is that the osmotic pressure gradient only regulates the transport of water (i.e., the water molecules could be diffused through the AQP along the osmotic pressure gradient) instead of the so-called “turn-on” or “turn-off” phases. Thus, water molecules could be directly allowed into and out of the cells. Once the endothelial cells are injured, the expression of AQP increases, which augments capillary permeability and is believed to be closely associated with the onset of hydrocephalus. To date, there are 13 kinds of proteins that have been identified as members of the AQP family, which include AQP0 to AQP12. These 13 *AQP* genes exert diversified physiological functions due to the different expression sites with similarities in permeability [34].

The transcellular pathways of water are distributed in all tissues but are mainly in epithelial and endothelial cells that have functions related to the secretion and the absorption of fluid. These proteins participate in the regulation of secreting and absorbing water as well as in the homeostasis between extra- and intracellular fluid volume.

Distributed in the outer medullary descending vasa-recta (OMDVR), the AQP1 is related to the mechanism of urinary concentration. *AQP1* ^−/−^ mice showed a deficiency in urinary concentration due to the damaged water transport pathway that was energized by osmotic pressure and exhibited symptoms such as polyuria, hypotonicity of the urine, decreased response to vasopressin, significantly enlarged OMDVR and adaptive capillary wall reconstruction.

The expression of AQPs is diversified based on different places in the lung, (e.g., the *AQP1* gene is mainly expressed in the capillary endothelium; the *AQP3* gene is mainly expressed in the epithelial cells of both the nasopharynx and the airway; the *AQP4* gene is mainly expressed in the basal-lateral membrane in the epithelial cells of the airway; and the *AQP5* gene is mainly expressed in the epithelial cells in the alveoli). Both the *AQP1* and the *AQP5* genes can regulate the osmotic water transport in the capillaries of the airway, but do not affect the humidification or the liquid homeostasis on the surface of the airways.

In the thoracic tissues, the *AQP1* gene is expressed in the endothelial cells of both the visceral and cervical pleura as well as the mesothelial cells in the cervical pleura to regulate the osmotic pressure balance of fluid inside and outside the thoracic cavity. Researchers have found that the *AQP1* gene is intimately associated with the extent of cardiac necrosis in cardiopulmonary bypass and infarction models, which suggests a potential relationship between the myocardial ischemia and the maintenance of myocardial edema [35].

Expressed by hepatic cells, the *AQP8* gene is mainly located in the cellular vesical. During the process of bile secretion, the *AQP8* gene in the membrane is transported to the canalicular membranes, which are induced by cAMP and increase the water permeability of the apical membrane, thus achieving water transport [36]. In addition, Drobná Z et al. [37] found that the bile secretion by bile canaliculi is synergistically regulated by the *AQP8* gene and hepatic sinusoidal *AQP9* gene, which mediates water transport between the hepatocytes and the blood in the hepatic sinusoidal endothelium. Distributed on the basal membrane of intrahepatic cholangiocytes, *AQP4* can maintain the equilibrium of water permeability between the apical area and the basal membrane of cholangiocytes and mainly regulates the water permeability of the basal membrane.

The permeability of the blood-brain barrier in brain tissues is also associated by some subtypes of *AQP*. Teng et al. [38] found a significant and positive correlation between the expression of *AQP4* mRNA and the permeability of the blood-brain barrier in mice after cerebral hemorrhage. Ke et al. [39] identified focal changes in the expression of *AQP4* mRNA in cases hydrocephalus with concomitantly damaged blood-brain barriers, while there were no significant changes in cases of hydrocephalus that were caused by diffuse brain injuries without any damage to the blood-brain barriers. In addition, the research by Liu et al. [40] indicated that overexpressed *AQP9* gene after a cerebral hemorrhage was closely associated with the damage of the blood-brain barrier and the formation of hydrocephalus. The possible explanatory mechanism is that, while in an ischemic and hypoxic environment, the Na^+^-K^+^-ATPase of the membrane in the brain tissues is destroyed and the balanced ion exchange in the membrane is broken, thus activating the expression of the *AQP9* gene, which perceives changes in osmotic pressure.

#### Vesicle

Under normal physiological conditions, the transport of bio-macromolecules in the blood is strictly restricted by the endothelial barrier, which makes the paracellular pathway only permeable to small molecular substances, such as glucose or blood urea nitrogen, while it is impossible for macromolecular substances like albumin to pass through the membrane directly. The transport of such macromolecular substances depends on the vesicles, a kind of cellular structure, by which the molecules awaiting transport will be absorbed and then delivered to the organelles of the target cell or extracellularly. This pathway plays a crucial role in maintaining the normal colloid osmotic pressure of the tissues. Plasmalemmal vesicles that include the caveolae and fenestrae are currently being studied.

Like the subcellular structure, the caveola is a flask-shaped, cave-like invagination specifically spread on the surface of the cytoplasmic membrane with a diameter that ranges from 50 to 100 nm. Caveolae are widely spread throughout various cells but are rich in endothelial, epithelial and smooth muscle cells with the existence of the Caveolin molecule as the representative feature [41]. Caveolae mainly participate in the endocytosis and pinocytosis of macromolecular substances and in signal transduction [42]. As a kind of integrin membrane protein with a molecular weight that ranges from 21 to 24 kD, caveolin plays important roles in maintaining the morphological integrity, structure and function. Currently identified members of caveolins family include caveolin-1ɑ, caveolin-1β, caveolin-2ɑ, caveolin-2β, caveolin-2γ and caveolin-3 [43]. Caveolin-1 and caveolin-2 are expressed in most cells (e.g., epithelial cells, endothelial cells, adipocytes, fibroblasts, smooth muscle cells, etc.), especially in the cells of the cardiovascular system, where the expression of caveolin-1 and caveolin-2 can be directly observed. However, caveolin-3 is specifically expressed in muscle cells (i.e., smooth muscle cells, skeletal muscle cells, cardiac muscle cells, astrocytes, chondrocytes, etc.) and is intimately associated with the synthesis of muscle cells [44].

Fenestrae, with a diameter of 107 ± 1.5 nm, are round-shaped, sieve plate-like, and clustered transcellular passages [45]. However, Xu et al. [46] showed that the diameter of fenestrae was proven to be 60–80 nm . Research has confirmed that these fenestrae account for 20–50% of the surface area of endothelial cells and act as the bi-directional guardian in the regulation of the transcellular passage for the transport of macromolecular substances such as albumin [46]. Under electron microscopy, Ryan et al. [47] found that the transport of macromolecular substances like albumin was mainly regulated by a layer of polysaccharide-protein complexes in the domain of fenestrae that were covered on the surface of the endothelial cells. These complexes included oligosaccharide chains of glycoproteins and proteoglycans with relative GAG, such as chondroitin sulfate and heparin sulfate [48]. In addition, the location of different fenestrae varies. Open fenestrae that lack any structural obstructions are mainly located in the thin peripheral cytoplasm while the complexes of multi-folded fenestrae are organized as labyrinthine structures that are found in the proximity of the perinuclear area [49]. Labyrinthine fenestrae constitute about one-third of the total porosity of endothelial cells.

## Signal mechanisms and major inducing factors of vascular leakage

### Signaling mechanisms of vascular permeability

Cell-cell junctions and cell-basal lamina junctions are all connected by cytoskeletal proteins. Variance in cytoskeletal functions can lead to changes in the adhesive states of intercellular and basal lamina cell junctions that can induce the rearrangement of skeletal proteins through the mechanism of signal transduction, which alters the permeability of endothelial cells.

**Fig. 1:**
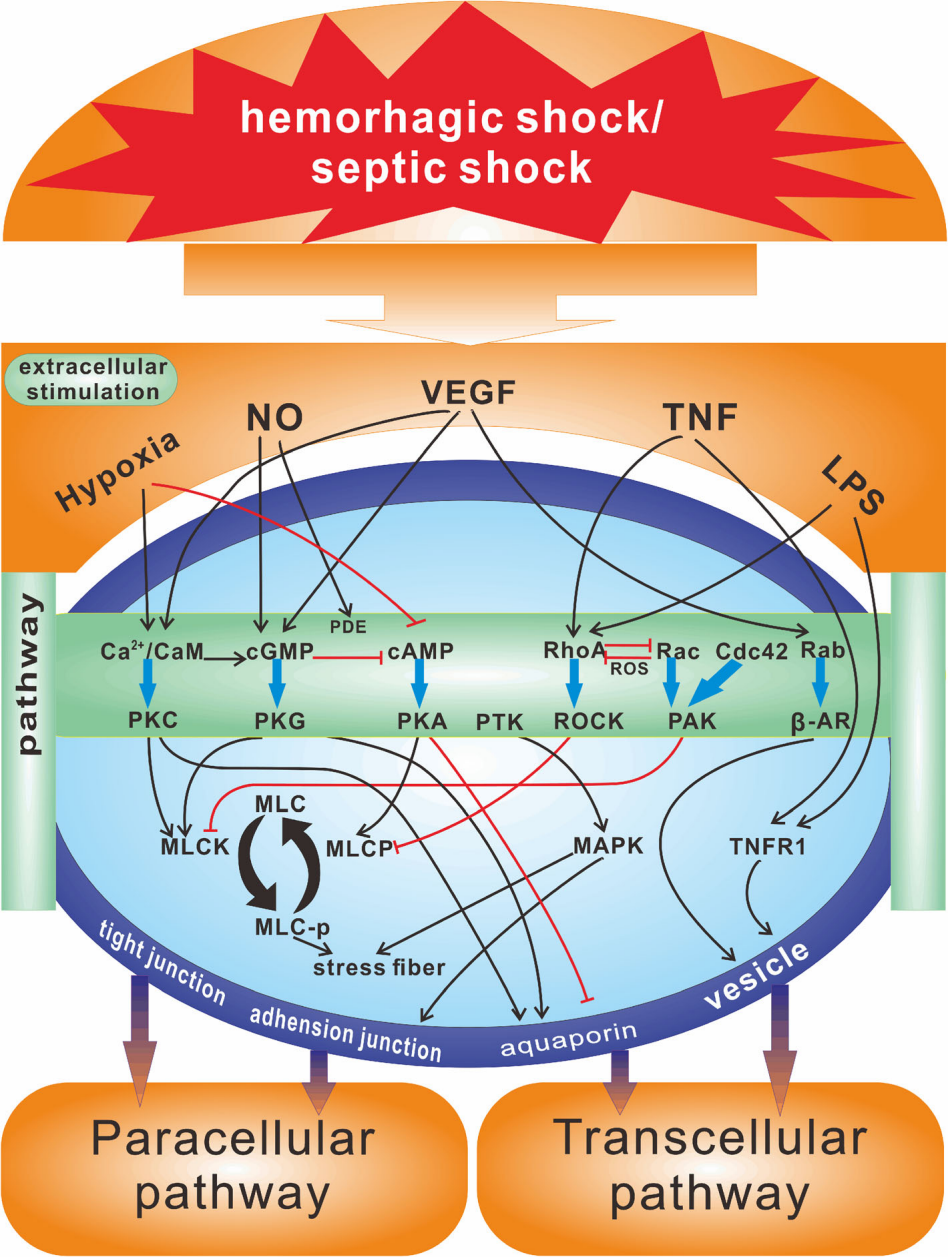
The mechanism of signaling transduction of vascular permeability. NO, Nitric Oxide; VEGF, vascular endothelial growth factor; TNF, Tumor necrosis factor; PKC, Protein kinase C; PDE, Phosphodiesterase; cGMP, Cyclic guanosine monophosphate; PKG, Protein kinase G; cAMP, Cyclic adenosine monophosphate; PKA, Protein Kinase A; PTK, Protein Tyrosine Kinase; RhoA, Ras homolog gene family member A; ROCK, Rho-associated coiled-coil-containing protein kinase; ROS, Reactive oxygen species; Rac, Ras-related C3 botulinum toxin substrate 1; PAK, p21-Activated Kinase; Cdc42, Cell division control protein 42; AR, Androgen receptor; MLC, Myosin light chain; MLCP, Myosin light chain phosphatase; MLCK, Myosin Light Chain Kinase; MAPK, Mitogen-activated protein kinase; TNFR1, Tumor necrosis factor receptor 1. a) Ca^2+^-PKC/CaM pathway, the vascular permeability is regulated in the tight junction by controlling the phosphorylation level of MLC by MLCK; b) cGMP-PKG pathway, the vascular permeability is regulated by *AQP* activity and tight junctions through controlling the phosphorylation level of MLC by MLCK; c) cAMP-PKA pathway, the vascular permeability is regulated by adjusting the activity of MLCP and *AQP*; d) PTK-MAPK pathway, the vascular permeability is affected by regulating the tight junctions by FAK; e) SGP pathway (small G protein), the vascular permeability is regulated by adjusting the tight junction, the adherens junctions and the vesicles by Rho and Rac GTPases and by CDC42

Research has indicated that actin filaments are presented in cells as filamentous actin, F-actin, and global actin, G-actin. Under normal physiological conditions, the actin filaments in endothelial cells are mainly spread in the nuclear and peripheral areas of cells and form a compact peripheral band. By activating the ATPase that is located in the globular head of the myosin heavy chain, the phosphorylated *MLC* gene can generate the energy needed to mobilize the F-actin filament in the actin cytoskeleton with a compact peripheral band of microfilaments that has disappeared. This process forms the stress fiber that is composed of a non-polar and single-rowed actin filament with altered cellular morphology, which results in enlarged and increased intercellular gaps and augmented permeability [50]. The MLCK directly transferred into the capillaries that are isolated for culture can induce a significant increase in endothelial cell permeability, while an MLCK inhibitor, such as ML-7 or BAPAT, can protect the functions of endothelium by suppressing the increase in EC permeability that is induced by thrombin. Research has confirmed that an increase in the permeability of the intestinal mucosal barrier at an early stage with the augmentation of protein expression of the MLCK and the phosphorylation of the MLC, specifically MLC-inhibited phosphorylation mediated by MLCK, can significantly reduce the increased amplitude in the permeability of the intestinal mucosal barrier and alleviate the tissue injuries and the changes in ZO-1 [51]. In addition, calyculin, the MLCP inhibitor, can significantly increase capillary permeability and simultaneously sustain the phosphorylation level of the *MLC* gene. Various signal pathways (Fig. 1) are involved in these regulatory mechanisms: a) the Ca^2+^-PKC/CaM pathway can regulate vascular permeability through modification of the tight junction by controlling the phosphorylation level of MLC by MLCK; b) the cGMP-PKG pathway can regulate vascular permeability by regulating AQP activity and tight junctions through controlling the phosphorylation level of MLC by MLCK; c) the cAMP-PKA pathway can regulate vascular permeability by adjusting the activity of MLCP and AQP; d) the PTK-MAPK pathway can affect vascular permeability by regulating the tight junctions by focal adhesion kinase (FAK); e) the small G protein (SGP) pathway can regulate vascular permeability by adjusting the tight junction, the adherens junctions and the vesicles through Rho and Rac GTPases and CDC42.

### Major inducing factors for vascular leakage

Signal transduction mechanisms affecting the permeability of the vascular endothelium are relatively complicated, and various inducing factors such as the inflammatory mediators of Ca^2+^, nitric oxide (NO), vascular endothelial growth factor (VEGF) and thrombin, can alter the junction-related cytoskeleton (e.g., actin and myosin), which further influences the contractile and morphological variations of endothelial cells.

#### VEGF

A type of glycogen, VEGF were first purified from the in vitro culture solution of astrocytes in the follicles of the pituitary gland and were named by Ferrara in 1989 [52]. VEGF can affect vascular permeability through several pathways. VEGF destroys the intercellular adherens junction and tight junction to increase vascular permeability and can promote the phosphorylation of Y658 and Y731, VE-cadherin, to destroy the mutual junction between VE-cadherin and β-catenin [53]. In addition, VEGF can break the intercellular tight junctions through the VEGFR-2 pathway, which precipitates vascular leakage [54–56]. Moreover, VEGF can increase vascular permeability by separating the intercellular junctions through the MAPK and PKC pathways. VEGF induces the formation of fenestrae and caveolae in the cytoplasm of endothelial cells to promote the penetration of the solute through the membrane. VEGF can induce the invagination of the membrane to form vesicles, which leads to an increase in fenestrae in the microtubular system in cells and the formation of penetrative gaps in the cytoplasm, which are extended from the integration of the luminal membrane to the basal lamina, which increases the vascular permeability of the endothelium [57, 58].

#### NO

NO can induce an increase in vascular permeability through two main ways. NO induces the cyclic guanosine monophosphate -protein kinase G (cGMP-PKG) signal pathway to increase vascular permeability, which is the most frequently seen pathway. Interactions between NO and soluble guanylyl cyclase (sGC) can induce the formation of cGMP to stimulate the *PKG* and the *MAPK* genes, thus increasing vascular permeability [59]. NO also increases vascular permeability through the S-Nitrosation on the sulfhydryl group of the relevant protein to destroy the adherens junctions, which increases vascular permeability. S-Nitrosation on the sulfhydryl group of the relevant protein is similar to phosphorylation and depends on some target proteins to stimulate or inhibit some biological processes [60]. Research has shown that variations in vascular permeability caused by S-Nitrosation correlate to the rapid decrease of the levels of two kinds of adherens junction proteins: β-catenin and p120-catenin [61, 62]. The increase of NO caused by tumor necrosis factor -α (TNF-α) and active platelet factors causes the upregulation of S-Nitrosation on the sulfhydryl group of the relevant protein.

#### Cytokines

Research has shown that interleukin and TNF play important roles in regulating shock-related vascular permeability. Persistent intravenous or intraperitoneal injections of IL-2 can cause “vascular leakage syndrome,” where extracellular fluid is rapidly accumulated and leads to ascites and pulmonary edema. In the latest research by Puerta-Guardo, the results indicated that significant increases in the expression of cytokines such as IL-6, IL-12, p70, TNF-α and prostaglandin E2 (PGE2) in septic shock could alter vascular permeability by destroying the tight junctions. Wiggins-Dohlvik et al. [63] found that TNF-α can separate the tight junctions by decomposing ZO-1 to increase vascular permeability.

#### Microparticles (MPs)

MPs refer to those vesicles generated by cells of 0.1 to 1 μm [64]. Lacking nuclei and the comprehensive ability of cells, MPs contain cytoskeletal proteins and are covered on the surface by certain amounts of phosphatidylserine [57]. MPs were once called “platelet dust” and were first discovered at the beginning stages of blood clotting. Current research has shown that MPs are not simply the production of invalid cellular fragments. The literature has confirmed that MPs could reduce vascular generation, promote oxidative stress, weaken vasodilatory effects and generate reactive oxygen species (ROS). Theoretically, MPs can be generated by all cells, which has been proven by some reports, but most studies believe that MPs originated from platelets, leukocytes and endothelial cells [65, 66]. Currently, there are many studies that are focusing on the origination of MPs from platelets. In recent years, the research has confirmed that MPs play an important role in promoting vascular leakage and maintaining equilibrium in homeostasis. The existing studies indicate that the MPs with cytokines or small RNA sequences can increase vascular permeability by acting on the vascular endothelium.

## New methods for the prophylaxis and treatment of vascular leakage

Methods for the prophylaxis and treatment of vascular leakage mainly include both conventional methods and newly developed targeted measures. Conventional methods include the correction of acidosis and glucocorticoid therapy, while the targeted measures for the variations in vascular permeability secondary to septic shock is performed on the inducing factors that cause the increase in vascular permeability and on the therapeutic targets. The main methods in this targeted approach include reducing the inducing factors and inhibiting the therapeutic targets to cause an increase in vascular permeability.

### Preventing vascular leakage by VEGF antagonism

Angiopoietin-1 (*Ang-1 or ANGPT1*) is a newly identified endogenous protein factor that can antagonize the vascular leakage caused by VEGF and inflammatory mediators. Gamble et al. (2000) indicated that ANGPT1 can offset approximately 70% of the vascular leakage caused by VEGF [67]. Thurston et al. (2000) found that the *ANGPT1* gene can stabilize the structure of the vascular wall can promote both the spread of endothelial cells and the formation of tuber-like structures [68]. Through specifically binding with TIE-2, the *ANGPT1* gene can cause the phosphorylation of its receptor and subsequent signal transmission. Removal of the *ANGPT1* and *TIE-2* genes can lead to vasodilation and increased vascular permeability, which is related to the activation process of FAK by the phosphatidylinositol 3-kinase (PI3K) [69].

Protein phosphatases 1 (PP1), as an effective Src-1 inhibitor that can significantly decrease vascular permeability. The increased expression of Src-1 and its phosphorylation were positively correlated to the levels of *VEGF* and were negatively correlated to the *ANGPT1* gene [70]. The inhibitory effect of PP1 on Src-1 may occur because of the downregulation of *VEGF* and the upregulation of the *ANGPT1* gene, which may further enhance the tight junctions and reduce vascular permeability [71].

### Reducing the vascular permeability by controlling the release of NO

Sphingosine-1-phosphate receptor 2 (S1pr2) can suppress the increase in shock-related vascular permeability by inhibiting the endothelial nitric oxide synthase (eNOS). Endothelial cells lacking S1pr2 exhibit severely damaged adherens junctions. Cui et al. [72] found that S1pr2 can protect the vessels by inhibiting the activity of protein kinase B (AKT), which is an activating enzyme for the eNOS. Previous studies on multiple kinds of cells indicated that the mammalian target of rapamycin (mTOR) can mediate the upregulation of the expression of the *VEGF* gene caused by hypoxia- inducible factor-1 alpha (HIF-1ɑ) and that the expression of mTOR is regulated by either PI3K/Akt or PKCδ. Choi et al. [73] found that rottlerin, a kind of inhibitor of PKCδ, can result in the suppression of the activity of the upstream signal of the PI3K/Akt of the *VEGF* gene to inhibit the expression of the mTOR-HIF-1a-VEGF pathway and increase vascular permeability.

In recent research, it has been proven that a new type of metal complex can also participate in the regulation of vascular permeability by controlling the release of NO. Monti et al. [74] identified the protective effect of Ni(PipNONO)Cl, a complex of Ni, in an experiment of in vitro cultures of vascular endothelial cells. When the cultured endothelial cells were exposed to IL-1β, the increase in vascular permeability was realized by the Ni(PipNONO)Cl, which suppressed the activity of cyclooxygenase-2 (COX-2) inhibitors to downregulate the expression of the inducible nitric oxide synthase (iNOS). Silva et al. [75] found that the Au nanoparticle (AuNPs) also regulated the release of NO and the arterial vasodilation in rats. In this study, a cluster formation that was generated by a combination of AuNPs and a ruthenium complex (Ru-4PySH), which is a donor of NO, reduced the release of NO and increased vascular permeability.

### Reducing vascular permeability by inhibiting the phosphorylation of MLC

Atrial natriuretic peptide (ANP) can reduce the endothelial leakage caused by injuries. Rho-specific guanine nucleotide exchange factors (GEF-H1) released by microtubules can stimulate the Rho pathway and increase vascular permeability, while ANP can be used specifically to treat pathological vascular leakage through regulating the function of GEF-H1. In addition, ANP can also inhibit the phosphorylation of the *MLC* gene by inducing the inactivation of the Rac-PAK1 dependent GEF-H1 to suppress the activity of the Rho pathway [76]. Interestingly, ANP can be rapidly degraded by neprilysin (NEP), which can be suppressed by *omapatrilat*, thus reducing the leakage area of the arteries. Ichiki et al. (2013) found that such a dual inhibitory effect on NEP might be related to the ANP/cGMP pathway [77]. Chen et al. [78] found that ANP can impede the phosphorylation of the *MLC* gene by inhibiting the GC/cGMP and the TRPC6 pathways. Such potential efficacy on vascular leakage was identified in an observational experiment that sildenafil (i.e., Viagra) could enhance the effect of ANP on the endothelium to improve the systemic endothelial barrier functions.

In addition, pertussis toxin can offset the decrease in the level of cAMP induced by hypoxia to reduce vascular leakage. Dexamethasone can also increase the level of intracellular-soluble cAMP to suppress the phosphorylation of the *MLC* gene, which enhances the barrier functions of endothelial cells. Maharjan et al. [79] found that Sac-1004 can increase the content of cAMP, activate the Rac pathway and improve the integrity of the endothelial cell to enhance the endothelial barrier functions.

### Preventing vascular leakage by protecting intercellular junctions

It has been recently confirmed that Wnt and its homologue, R-spondin, can also be used to treat inflammation-related conditions in addition to the regulation of embryonic development. Kannan et al. [80] found that R-spondin3 (R-spo3) can maintain the integrity of endothelial cell barriers by enhancing the junctions between endothelial cells and preserving the positions of VE-cadherin and F-actin in the cellular peripheral zone. It has been confirmed that both inflammation and apoptosis secondary to subarachnoid hemorrhage (SAH) are of significance because of increases in vascular permeability. Zhou et al. [81] indicated that urinary trypsin inhibitors can exert anti-inflammatory and anti-apoptotic effects by inhibiting the activity of the c-Jun N-terminal kinase (*JNK*), nuclear factor-kappa B (*NF-кB*) and *p53* genes to slow the increase in vascular permeability secondary to SAH. Moreover, diosmin also plays an important role in maintaining the integrity of intercellular junctions and reducing vascular permeability as a kind of natural flavonoid glycoside. An experiment was performed to detect the interruption of the blood-retina barrier by Evans blue staining and assaying the structural variations in tight junctions by transmission through an electron microscope. Tong et al. [82] found that diosmin can increase the content of ZO-1 and occludin in tight junctions and can maintain the integrity of tight junctions to prevent an increase in vascular permeability, which showed the protective effect on retinal capillaries after ischemic reperfusion.

## Conclusions

Although an increase in vascular permeability has been proven to be necessary in various normal physiological processes, such as embryonic vasculogenesis, formation of the menstrual cycle and courses of wound-healing, pathological increases in vascular permeability will result in an increase of colloid osmotic pressure of the interstitial space, which can seep liquid into the thoracic and abdominal cavities and create a decrease in the effective circulating blood volume. Meanwhile, various degrees of leakage that occur in the lung can lead to hypoxemia, which can further induce tissue hypoxia, creating a vicious cycle that finally results in multiple organ dysfunction syndrome. Therefore, it is of great significance to determine the most effective therapy for the treatment of pathologically increased vascular permeability.

The current methods of prevention focus mainly on the regulation of paracellular and transcellular signaling pathways or by blocking related inducing factors. Our research recently found that the release of microparticles and the membrane-protective mechanisms may also play important roles in the prevention of vascular leakage. Specific regulatory mechanisms are currently being researched, which will likely provide new therapeutic targets for the treatment of vascular diseases. However, we must realize that most current vascular leakage therapies target either single signal or inducing factors, and comprehensively treating vascular leakage to improve organic circulatory functions after shock may be the future challenge regarding the management of vascular permeability.

## Abbreviations

AKT: Protein kinase B
ANGPT1: Angiopoietin-1
ANP: Atrial natriuretic peptide
AQP: Aquaporin
ARDS: Acute respiratory distress syndrome
AuNPs: Au nanoparticle
cGMP: Cyclic guanosine monophosphate
CLS: Capillary leak syndrome
COX-2: Cyclooxygenase-2
ERK: Extracellular signal-regulated kinase
FAK: Focal adhesion kinase
Flk-1: Fetal liver kinase 1
GEF-H1: Rho-specific guanine nucleotide exchange factors
HIF-1ɑ: Hypoxia- inducible factor-1 alpha
ICU: Intensive care units
iNOS: Inducible nitric oxide synthase
JAM: Junction adhesion molecule
JNK: c-Jun N-terminal kinase
MAPK: Mitogen-activated protein kinase
MLCK: Myosin light chain kinase
MLCP: Myosin light chain phosphatase
MODS: Multiple organ dysfunction syndrome
MODS: Multiple organ dysfunction syndromes
MPs: Microparticles
mTOR: Mammalian target of rapamycin
NEP: Neprilysin
NF-кB: Nuclear factor-kappa B
NO: Nitric oxide
OMDVR: Outer medullary descending vasa-recta
PGE2: Prostaglandin E2
PI3K: Phosphatidylinositol 3-kinase
PKC: Protein kinase C
PKG: Protein kinase G
PP1: Protein phosphatases 1
ROS: Reactive oxygen species
R-spo3: R-spondin3
S1pr2: Sphingosine-1-phosphate receptor 2
SAH: Subarachnoid hemorrhage
sGC: Soluble guanylyl
SGP: Small G protein
TEER: Trans-epithelial electrical resistance
TNF-α: Tumor necrosis factors -α
VE-cadherin: Vascular endothelial- cadherin
VEGF: Vascular endothelial growth factors
VVOs: Vesicular-vacuolar organelles
ZO: Zona occludens
